# *BMC Cancer* reviewer acknowledgement 2015

**DOI:** 10.1186/s12885-016-2151-2

**Published:** 2016-02-15

**Authors:** Dafne Solera

**Affiliations:** BioMed Central, Floor 6, 236 Gray’s Inn Road, London, WC1X 8HB, UK

## Abstract

The editors of BMC Biotechnology would like to thank all our reviewers who have contributed to the journal in Volume 15 (2015).

**Mohammed Abba**

Germany

**Abbas Abbas**

USA

**Daniel Abbott**

USA

**Rym Abderrahmane**

Algeria

**Takashige Abe**

Japan

**Thomas Abrams**

USA

**Jose Abreu**

Brazil

**Gudrun Absenger**

Austria

**Filippo Acconcia**

Italy

**Maria Isabel Achatz**

Brazil

**B.R. Achyut**

USA

**Maribel Acién**

Spain

**Jennifer E. Adair**

USA

**Agnieszka Adamczyk**

Poland

**Guy Adami**

USA

**Sarah Adams**

USA

**Vahid Afshar**

USA

**Patricia Agaba**

Nigeria

**Subhash Mohan Agarwal**

India

**Luca Agnelli**

Spain

**Nishant Agrawal**

USA

**Nadine Aguilera**

USA

**Mark Agulnik**

USA

**Golo Ahlenstiel**

Australia

**Faiz Ahmad**

USA

**Myung-Ju Ahn**

South Korea

**Haseeb Ahsan**

India

**Jiang Ai-Gui**

China

**Arihrio Aihara**

Japan

**Kiwamu Akagi**

Japan

**Naoki Akanuma**

USA

**Esra Akbay**

USA

**Tomi Akinyemiju**

USA

**Bilge Aktas**

USA

**Laura Alaniz**

Argentina

**Scott Albert**

USA

**Megan Albertelli**

USA

**Saverio Alberti**

Italy

**Cheryl Albright**

USA

**Kevin Albuquerque**

USA

**Thierry Alcindor**

Canada

**Jennifer Aldrink**

USA

**Fares Al-Ejeh**

Australia

**Krasimira Aleksandrova**

Germany

**Ramon Alemany**

Spain

**Mohammed Aleskandarany**

UK

**Riley Alexander**

USA

**Holly Algood**

USA

**Belal Al-Husein**

Jordan

**Abdelkrim Alileche**

USA

**Noorjahan Banu Alitheen**

Malaysia

**Azra Alizad**

USA

**Mohamad Allaf**

USA

**Alison Allan**

Canada

**Brian Allen**

USA

**Victoria Allgar**

UK

**Martin Almquist**

Sweden

**Lorenzo Alonso**

Spain

**Katherine Alpaugh**

USA

**Lutz Altenhofen**

Germany

**Deborah Altomare**

USA

**Begona Alvarez-Gonzalez**

USA

**Michelle Alves**

Brazil

**Mutsuki Amano**

Japan

**Frederic Amant**

Belgium

**Behrang Amini**

USA

**Steve Amireault**

USA

**Roland Ammann**

Switzerland

**Vito Amoroso**

Italy

**L. Amundadottirl**

USA

**Junhui An**

China

**Satoshi Anai**

Japan

**Venkateshwari Ananthapur**

India

**Claus Andersen**

Denmark

**Lesley Anderson**

UK

**Claudia Andl**

USA

**Nicolaus Andratschke**

Germany

**Carol Andrews**

USA

**Christina Angeles**

USA

**Sabrina Angelini**

Italy

**Eva Angenete**

Sweden

**Melinda Angus-Hill**

USA

**Laura Annaratone**

Italy

**Christina Annunziata**

USA

**Amélie Anota**

France

**Morad Ansari**

UK

**Ahti Anttila**

Finland

**Smith Apisarnthanarax**

USA

**Tanya Applegate**

Australia

**Leonard Appleman**

USA

**Yasuhito Arai**

Japan

**Marc Arbyn**

Belgium

**John Arcaroli**

USA

**Meritxell Arenas**

Spain

**Paolo Aretini**

Italy

**Frank Arfuso**

Australia

**Takaaki Arigami**

Japan

**Yoshiaki Arimura**

Japan

**Elif Damla Arisan**

Turkey

**Guillermo Armaiz Pena**

USA

**Andrew Armstrong**

USA

**Kristan Aronson**

Canada

**Shilpi Arora**

USA

**Arantzazu Arrospide**

Spain

**Eishi Ashihara**

Japan

**Cheryl Ashworth**

UK

**Laura Asnaghi**

USA

**Marie-Liesse Asselin-Labat**

Australia

**Smita Asthana**

India

**Paulita Pamela Astudillo**

Philippines

**Beste M. Atasoy**

Turkey

**Michael Atkinson**

Germany

**Katarzyna Augoff**

Poland

**Holger Auner**

UK

**Stefanie Aust**

Austria

**Romane Auvergne**

USA

**Ka Wai Au-Yeung**

Hong Kong

**Amir Avan**

Netherlands

**Margaritis Avgeris**

Greece

**A. Ávila-Flores**

Spain

**Eli Avisar**

USA

**Musaddiq Awan**

USA

**Niranjan Awasthi**

USA

**David Axelrod**

USA

**Ali Azhdarinia**

USA

**Hamdy Azim**

Egypt

**Syed Aziz**

Canada

**Ebrahim Azizi**

USA

**Masoud Azodi**

USA

**Hideo Baba**

Japan

**Jean-Baptiste Bachet**

France

**Floor Backes**

USA

**Shahed Badiyan**

USA

**Doaa Badr**

Egypt

**Ellena Badrick**

UK

**Saleh Baeesa**

Saudi Arabia

**Moo Jun Baek**

South Korea

**Maria Baggstrom**

USA

**Marina Bagnoli**

Italy

**Amit Bahl**

UK

**Shahram Bahmanyar**

Sweden

**S.B. Bai**

China

**Anamika Bajpai**

USA

**Martin Bak**

Denmark

**Christopher Bakkenist**

USA

**Debora Balabram**

Brazil

**Francesc Balaguer**

Spain

**Kumudha Balakrishnan**

USA

**David Balayssac**

France

**Gianluca Baldanzi**

Italy

**Enoch Baldwin**

USA

**Marija Balic**

Austria

**Balint László Balint**

Hungary

**Justin Balko**

USA

**Fatima Baltazar**

Portugal

**Richard Bambury**

Ireland

**Rameshwar Bamezai**

India

**Aristotle Bamias**

Greece

**Oluwaseun Adebayo Bamodu**

Taiwan

**Jesus Banales**

Spain

**Vimla Band**

USA

**Sourav Bandyopadhyay**

USA

**Anita Bane**

Canada

**Sulagna Banerjee**

USA

**Versha Banerji**

Canada

**Aria Baniahmad**

Germany

**Malgorzata Banys**

Germany

**Yongping Bao**

UK

**Fedricker Barber**

USA

**Maria Barbolina**

USA

**Andrew Barbour**

Australia

**Jo Barnes**

New Zealand

**Martin Barr**

Ireland

**Simon Barry**

Australia

**Rachel Bar-Shavit**

Israel

**Sarah Bass**

USA

**Elen Bastos**

Brazil

**Anupam Basu**

India

**Nicholas Bateman**

USA

**Giacomo Batignani**

Italy

**Martin Baumgartner**

Switzerland

**Dirk Bausch**

Germany

**Viviana Bazan**

Italy

**Francisco Beca**

USA

**Tim Beck**

USA

**Daniel Becker**

USA

**Jennifer Beebe-Dimmer**

USA

**Alicia Beeghly-Fadiel**

USA

**Andre Beer-Furlan**

USA

**Fariba Behbod**

USA

**Gundula Behrens**

Germany

**James Bekeny**

USA

**Antonino Belfiore**

Italy

**Martin Belinsky**

USA

**Katy Bell**

Australia

**Marcia Bellon**

USA

**Al Benson**

USA

**Ronald Benveniste**

USA

**Matthias Benz**

Switzerland

**Sebastien Benzekry**

France

**Plinio Berardo**

Brazil

**James Berenson**

USA

**Andrew Bergen**

USA

**Thorsten Berger**

Canada

**Christine Bergeron**

France

**Lothar Bergmann**

Germany

**Emerson Bernardes**

Brazil

**Dominique Bernard-Gallon**

France

**Henri Bernardi**

France

**Kimberly Bertrand**

USA

**Benoit Beuselinck**

Belgium

**John Beutler**

USA

**Philippe Beuzeboc**

France

**Kishor Bhakat**

USA

**Amit Bhargava**

USA

**Sanjay Kumar Bharti**

India

**Natarajan Bhaskaran**

USA

**Krishna Bhat**

USA

**Vijaya Bhatt**

USA

**Bhaskar Bhattacharya**

Singapore

**Debmalya Bhattacharyya**

USA

**Ujjal Bhawal**

Japan

**Sankarprasad Bhuniya**

India

**Roberto Bianco**

Italy

**Hemant Kumar**

Bid USA

**Francois-Clement Bidard**

France

**Jana Biermann**

Sweden

**Barbara Biesecker**

USA

**Claudia Bighin**

Italy

**Irene Bijnsdorp**

Netherlands

**Jacek Bil**

Poland

**Caroline Billingsley**

USA

**Muhammad Shahdaat Bin Sayeed**

Bangladesh

**Lynne Bingle**

UK

**Vincent Biron**

Canada

**Faraz Bishehsari**

USA

**Dr Bishop-Bailey**

UK

**Bivas Biswas**

India

**Esther Black**

USA

**David Blackbourn**

UK

**Eric Blair**

UK

**George Blanck**

USA

**Carmen Blanco-Aparicio**

Spain

**Giovanni Blandino**

Italy

**Susanne Blank**

Germany

**Josip Blonder**

USA

**Fiona Blows**

UK

**Christina Bluemel**

Germany

**Hubert Blum**

Germany

**Scott Blume**

USA

**Miroslav Blumenberg**

USA

**Gerd Bobe**

USA

**Sharon Bober**

USA

**Maurizio Bocchetta**

USA

**Kristien Boelaert**

UK

**Hans Bogaards**

Netherlands

**Mogens Karsbøl Boisen**

Denmark

**Patrick Boland**

USA

**Renzo Boldorini**

Italy

**Azam Bolhassani**

Iran

**Subhashini Bolisetty**

USA

**Laura Bonanno**

Italy

**Benjamin Bonavida**

USA

**Jesper Bonde**

Denmark

**Alois Bonifacio**

Italy

**Marcelo Bonomi**

USA

**Carolina Bonzi**

Italy

**Guntram Borck**

Germany

**Louise Bordeleau**

Canada

**Michael Bordonaro**

USA

**Wegene Borena**

Austria

**Ulrich Bork**

Germany

**Carl Bortner**

USA

**Francis Boscoe**

USA

**Remko Bosgraaf**

Netherlands

**Fred Bosman**

Switzerland

**Paolo Bossi**

Italy

**Gwenola Boulday**

France

**Anicet Luc Magloire Boumba**

Congo

**Aurelie Bourmaud**

France

**Hassan Bousbaa**

Portugal

**Wilbur Bowne**

USA

**Brian Boyce**

USA

**Mark Boyd**

UK

**Vildan Bozok Cetintas**

Turkey

**Marques Bradshaw**

USA

**Danielle Braggio**

USA

**Claudio Brancolini**

Italy

**Giovanni Brandi**

Italy

**Dana Brantley-Sieders**

USA

**Martin Braun**

Germany

**Luka Brcic**

Austria

**Anders Bredberg**

Sweden

**W. Nathaniel Brennen**

USA

**Darren Brenner**

Canada

**James Derek Brenton**

UK

**Patrick Brest**

France

**Kate Brettingham-Moore**

Australia

**Constance Brinckerhoff**

USA

**Nana Brochmann**

Denmark

**Guy Brock**

USA

**Nigel Brockton**

Canada

**Alexander Brodsky**

USA

**Jonathan Brody**

USA

**James Brooks**

USA

**Richard Brown**

USA

**Isaac Brownell**

USA

**Sara Yvonne Brucker**

Germany

**Wolfgang Brückl**

Germany

**Adam Brufsky**

USA

**Øyvind Bruland**

Norway

**Matteo Brunelli**

Italy

**Francesca Bruzzese**

Italy

**Richard Bryant**

UK

**Tomas Buchler**

Czech Republic

**Michael Buckstein**

USA

**Eva Budinska**

Czech Republic

**Mateusz Bujko**

Poland

**Joanna Burdette**

USA

**Mark Burkard**

USA

**Timothy Burns**

USA

**Bryan Burt**

USA

**Benedetta Bussolati**

Italy

**Anita Zarina Bustam**

Malaysia

**Lill-Tove Busund**

Norway

**Sebastiano Buti**

Italy

**Salma Butt**

Sweden

**Asta Bye**

Norway

**Jennifer Byrne**

Australia

**Nicholas Cacalano**

USA

**Carme Caelles**

Spain

**Mary Cahill**

Ireland

**H. Cai**

China

**Sabine Caillaud**

France

**Saverio Caini**

Italy

**Gregory Calip**

USA

**Natalie Callander**

USA

**Silke Cameron**

Germany

**E. Ramsay Camp**

USA

**Luca Campana**

Italy

**Belinda Campbell**

Australia

**Maria Campos**

Brazil

**Ferdinando Carlo Maria Cananzi**

Italy

**Silvana Canevari**

Italy

**Miguela Ayala Caniza**

USA

**Robert Canter**

USA

**Wei Cao**

China

**Melania Capasso**

UK

**Jaume Capdevila**

Spain

**Marzia Capelletti**

USA

**Giovanni Capone**

Italy

**Vera Cappelletti**

Italy

**Carmine Carbone**

Italy

**James Cardelli**

USA

**Alex Cardenas**

USA

**Amancio Carnero**

Spain

**Susan Carozza**

USA

**Francesca Carozzi**

Italy

**Philip Carpenter**

USA

**Darren Carpizo**

USA

**Shamus Carr**

USA

**Hetty Carraway**

USA

**Jason Carroll**

UK

**Bendix Carstensen**

Denmark

**Marc Cartellieri**

Germany

**Joana Carvalho**

Portugal

**Pilar Carvallo**

Chile

**Silvia Casadei**

USA

**Michael Cascio**

USA

**Luciano Cascione**

Switzerland

**Ryan Cassaday**

USA

**Andrea Cassidy-Bushrow**

USA

**Gemma Castaño-Vinyals**

Spain

**Maria Castella**

USA

**Rogerio Castilho**

USA

**Mª Angeles Castilla Moro**

Spain

**Gabriella Castoria**

Italy

**Javier S. Castresana**

Spain

**Magdalena Castro**

Chile

**Carlos Caulin**

USA

**Simon Cawthorn**

UK

**Ornella Cazzalini**

Italy

**Wim Ceelen**

Belgium

**Paloma Cejas**

Spain

**Maria Cekanova**

USA

**Ismail Çelik**

Turkey

**Chiara Alessandra Cella**

Italy

**Alessandra Ceolin Schmitt**

USA

**Matteo Cescon**

Italy

**Amphun Chaiboonchoe**

United Arab Emirates

**Arthit Chairoungdua**

Thailand

**Anindita Chakrabarty**

India

**Souvik Chakraborty**

USA

**Michael Chan**

Taiwan

**Vishal Chandra**

India

**Pi-Ling Chang**

USA

**Liu Chao**

China

**Caroline Chapman**

UK

**Al Charest**

USA

**Aditi Chatterjee**

India

**Arun Chauhan**

USA

**Alcides Chaux**

Paraguay

**Mariana Chavez Mac Gregor**

USA

**Harvey Checkoway**

USA

**Oren Cheifetz**

Canada

**Chang-Han Chen**

Taiwan

**Alfred Cheng**

Hong Kong

**Inna Chervoneva**

USA

**Siu Tim Cheung**

Hong Kong

**Eric Chevet**

France

**Ravindresh Chhabra**

India

**Dennis Chi**

USA

**Wei-Tso Chia**

Taiwan

**Minhsien Chiang**

Taiwan

**Chih-Yen Chien**

Taiwan

**Yoshitomo Chihara**

Japan

**Ludmilla Chinen**

Brazil

**Sinto Chirackal**

USA

**Tai-Jan Chiu**

Taiwan

**Je-Yoel Cho**

South Korea

**Yoon-La Choi**

South Korea

**Ravi Chokshi**

USA

**Yu-Ching Chou**

Taiwan

**Manish Chourasia**

India

**Balram Chowbay**

Singapore

**Vivek Chowdhary**

USA

**Edwin Choy**

USA

**Brock Christensen**

USA

**Peer Christiansen**

Denmark

**Mindy Christianson**

USA

**Dake Chu**

China

**Saranya Chumsri**

USA

**Benjamin Chung**

USA

**Michael Chuong**

USA

**Anil Chuturgoon**

South Africa

**Jennifer Chuy**

USA

**Raffaele Ciampi**

Italy

**Francesca Ciccarelli**

UK

**Joseph Ciccolo**

USA

**Savino Cilla**

Italy

**Kristen Ciombor**

USA

**Carlos J. Ciudad**

Spain

**Ludovica Ciuffreda**

Italy

**Emilio Ciusani**

Italy

**Jean Clairambault**

France

**Clancy Clark**

USA

**Marla Clayman**

USA

**Andrew Clayton**

Australia

**Brian Clem**

USA

**Aine Clements**

USA

**Mark Clendenning**

Australia

**Anne-Marie Cleton-Jansen**

Netherlands

**Gary Clifford**

France

**Marie Cohen**

Switzerland

**Monica Cojoc**

Germany

**Nananda Col**

UK

**William Coleman**

USA

**Tiago Collares**

Brazil

**Lara Collier**

USA

**Alison Colquhoun**

Brazil

**Anthony Conley**

USA

**Linda Connelly**

USA

**Roisin Connolly**

USA

**Vincenza Conteduca**

Italy

**Katherine Cook**

USA

**Colin Cooper**

UK

**Michael Coory**

Australia

**Mhairi Copland**

UK

**Rob Coppes**

Netherlands

**Ian Copple**

UK

**Jennifer Corcoran**

Canada

**Robert Cormier**

USA

**Paul Corn**

USA

**Cees Cornelisse**

Netherlands

**Giacomo Corrado**

Italy

**Simon Corrie**

Australia

**Elva Cortes**

Mexico

**Giuliana Cortese**

Italy

**Victoria Cortessis**

USA

**Giacomo Cossa**

Germany

**Nicholas Cost**

USA

**Bruno Costa**

Portugal

**Antonio Costanzo**

Italy

**Stefan Costinean**

USA

**Jocelyn Cote**

USA

**Travis Cotton**

USA

**Elliot Coups**

USA

**Peter Coussons**

UK

**Jermaine Coward**

Australia

**Thomas Cox**

Denmark

**Susanna Cramb**

Australia

**Joanne Crawford**

Canada

**Kim Creek**

USA

**Chiara Cremolini**

Italy

**Katherine Crew**

USA

**Sabrina Croce**

France

**Alysha Croker**

Canada

**Deirdre Cronin-Fenton**

Denmark

**David Crotzer**

USA

**Sheila Crowe**

USA

**Margaret Cruickshank**

UK

**Ofelia Cruz**

Spain

**Salvador Cruz-Flores**

USA

**Zobeida Cruz-Monserrate**

USA

**Alessandro Cucchetti**

Italy

**Bruce Cuevas**

USA

**Zoran Culig**

Austria

**Kevin Cullen**

USA

**Michele Cummings**

UK

**Julie Cunningham**

USA

**Margaret Currie**

New Zealand

**Karen Curtin**

USA

**Olivier Cuvillier**

France

**Marek Cybulski**

Poland

**Artur Czekierdowski**

Poland

**Brian J. Czerniecki**

USA

**Rakefet Czerninski**

Israel

**Anna Dabrowska-Iwanicka**

Poland

**Mariusz Dabrowski**

Poland

**Ramona Dadu**

USA

**Roi Dagan**

USA

**Kimberly Dahlman**

USA

**Donghai Dai**

USA

**Maria Grazia Daidone**

Italy

**Adrien Daigeler**

Germany

**Emanuele Dainese**

Italy

**Maria Dalamaga**

Greece

**Ashraf Dallol**

Saudi Arabia

**Benedict Daly**

USA

**Elisa Dama**

Italy

**Giovanna Damia**

Italy

**Nadia Dandachi**

Austria

**Daniela D’Angelo**

Italy

**Sophia L. Danhof**

Germany

**Daniel Danila**

USA

**Philippa Darbre**

UK

**Bhudev Das**

India

**Emilia Dauway**

Australia

**Heena Dave**

USA

**Lester Davids**

South Africa

**Ben Davidson**

Norway

**Ian Davis**

Australia

**Michelle Dawson**

USA

**Prithwish De**

Canada

**Pasqualino De Antonellis**

Italy

**Michele De Bortoli**

Italy

**Kevin De Braganca**

USA

**Daniel De Carvalho**

Canada

**Pierre De Delva**

USA

**An De Groef**

Belgium

**Steven De Jong**

Netherlands

**Ramon Andrade De Mello**

Portugal

**Jeroen De Ridder**

Netherlands

**Paul De Souza**

Australia

**Lise De Strooper**

Netherlands

**Marco De Velasco**

Japan

**Heather De Vries McClintock**

USA

**Silvia Deaglio**

Italy

**Kevin Debraganca**

USA

**Philip Debruyne**

Belgium

**Yusuf Deeni**

UK

**Norah Defamie**

France

**Olivier Dejardin**

France

**Tja Dekker**

Netherlands

**Rodney Dekoter**

Canada

**Egidio Del Fabbro**

USA

**Loic Deleyrolle**

USA

**Michel Delforge**

Belgium

**Hakan Demirci**

USA

**Binnaz Demirkan**

Turkey

**Gerard Den Heeten**

Netherlands

**Crystal S. Denlinger**

USA

**John Densmore**

USA

**Cheryl Der Ananian**

USA

**Philippe Deron**

Belgium

**Matthias Dettmer**

Switzerland

**Elisabeth Dexter**

USA

**Punita Dhawan**

USA

**Haryana Dhillon**

Australia

**John Di Guglielmo**

Canada

**Giuseppe Di Lorenzo**

Italy

**Massimo Di Maio**

Italy

**Parmeswaran Diagaradjane**

USA

**Maria Diakonova**

USA

**Dorota Diakowska**

Poland

**Patrícia Dias Pereira**

Portugal

**Teresita Díaz De Ståhl**

Sweden

**Mark Dickson**

USA

**Brenda Diergaarde**

USA

**Jennifer Dietrich**

USA

**Analisa Difeo**

USA

**Raghunadharao Digumarti**

India

**Ashok Dilly**

USA

**Helen Dimaras**

Canada

**Gabriel Dimattia**

Canada

**Helen Dimitriou**

Greece

**Christina Dimopoulou**

Germany

**Goberdhan Dimri**

USA

**Roberto Dina**

UK

**Michaela Dinan**

USA

**Keyue Ding**

Canada

**Daniela Dinulescu**

USA

**Darrell Dinwiddie**

USA

**Caroline Diorio**

Canada

**Richard Dipaolo**

USA

**Luc Dirix**

Belgium

**Gillian Dite**

Australia

**Thomas Dittmar**

Germany

**Dan Dixon**

USA

**Yavuz Dodurga**

Turkey

**Utku Dogan**

Turkey

**Jennifer Doherty**

USA

**Yoshihiro Dohi**

Japan

**Lianne Dolan**

Canada

**Corinne Doll**

Canada

**Joshua Doloff**

USA

**Ramona Dolscheid-Pommerich**

Germany

**Eytan Domany**

Israel

**Heymann Dominique**

UK

**Robert Domitrovic**

Croatia

**Peixin Dong**

Japan

**Monique Dontenwill**

France

**Rupa Doppalapudi**

USA

**Joanne Dorgan**

USA

**Oliver Dorigo**

USA

**Ken Dornfeld**

USA

**Emma Dorris**

Ireland

**Efrat Dotan**

USA

**Sinisa Dovat**

USA

**Jacob Dowden**

USA

**Jonathan Dowell**

USA

**James Downar**

Canada

**Amy Downing**

UK

**James Dowty**

Australia

**Gerald Doyle**

USA

**Bettina Drake**

USA

**Inge Driesprong-De Kok**

Netherlands

**Xiang Du**

China

**Shiwei Duan**

China

**Antonio Duarte**

Portugal

**M Joseph Duffy**

Ireland

**Anita Dunbier**

New Zealand

**Michael Duncan**

USA

**Neal Dunlap**

USA

**Philip Dunne**

UK

**Janice Dutcher**

USA

**Amit Dutt**

India

**Susan Dutton**

UK

**Ken Dutton-Regester**

Australia

**Laurence Duvillard**

France

**Michael Dwinell**

USA

**Lars Dyrskjot**

Denmark

**Peter Dziegielewski**

USA

**Helena Earl**

UK

**Hariharan Easwaran**

USA

**David Eberhard**

USA

**Lynn Eberly**

USA

**Lydia Eccersley**

UK

**Iris Eder**

Austria

**Richard Edmondson**

UK

**Melanie Edwards**

USA

**Fabio Efficace**

Italy

**Kristi Egland**

USA

**Brian Egleston**

USA

**Ann Marie Egloff**

USA

**Matthias Eiber**

Germany

**Jens Eickhoff**

USA

**Margret Einarson**

USA

**Eric Eisenhauer**

USA

**T.S. Karin Eisinger-Mathason**

USA

**Elizabeth Eklund**

USA

**Tomas Ekström**

Sweden

**Aya El Helali**

UK

**Samer El-Dika**

USA

**Miriam Elfström**

Sweden

**Daniel Elias**

Ethiopia

**Aristides Eliopoulos**

Greece

**Rossella Elisei**

Italy

**Lorraine Elit**

Canada

**Moshe Elkabets**

Israel

**Lesley Ellies**

USA

**Darrell Ellsworth**

USA

**Adam Elnaggar**

USA

**Mahmoud El-Readi**

Saudi Arabia

**Mohamed El-Shinawi**

Egypt

**Ashkan Emadi**

USA

**Cynthia Emory**

USA

**Makoto Emoto**

Japan

**Liliana Endo-Munoz**

Australia

**Kevin Hasegawa Eng**

USA

**Moulay Mustapha Ennaji**

Morocco

**Stefan Enroth**

Sweden

**Meira Epplein**

USA

**Joel Epstein**

USA

**Ane Gerda Eriksson**

Norway

**Carl Ernst**

Canada

**Hugo Escobar**

Germany

**Luis Mariano Esteban**

Spain

**Magdalena Esteva**

Spain

**David Euhus**

USA

**Andrea Evangelista**

Italy

**Gareth Evans**

UK

**Will Eward**

USA

**Obinna Ewkunife**

Germany

**Eduardo Eyras**

Spain

**Francesco Fabbri**

Italy

**Anthony C. Faber**

USA

**Carol Fabian**

USA

**Andrew Fabiano**

USA

**Tom Fahey**

Ireland

**Richard Fahlman**

Canada

**Anna-Karin Falck**

Sweden

**Stephen Falk**

UK

**Lifang Fan**

China

**Carolyn Fang**

USA

**Lucyana Farias**

Brazil

**Michelle Farkas**

USA

**Jeffrey Farma**

USA

**Ammad Farooqi**

Pakistan

**Alex Farr**

Austria

**Irfan Farukhi**

USA

**Elvira Favoino**

Italy

**Francesco Fazi**

Italy

**Tanja Fehm**

Germany

**Michael Feigin**

USA

**Jonas Feilchenfeldt**

Switzerland

**Marlena Fejzo**

USA

**Brunhilde Felding**

USA

**Emanuela Felley-Bosco**

Switzerland

**Wolfgang Fendler**

Germany

**Yibin Feng**

Hong Kong

**Francesco Feo**

Italy

**Sandra Fernandez**

USA

**Javier Fernandez-Ruiz**

Spain

**Manuela Ferracin**

Italy

**Lígia Ferreira**

Brazil

**Gustavo Ferrín**

Spain

**Anna Ida Fiaschi**

Italy

**Nicholas Fidelman**

USA

**Mary Jo Fidler**

USA

**Timothy A. Fields**

USA

**Steven Fiering**

USA

**Nicoletta Filigheddu**

Italy

**Cristina Fillat**

Spain

**Christine Fillmore**

USA

**Jorge Filmus**

Canada

**Mehdi Fini**

USA

**Neil Finkler**

USA

**Graeme Finlay**

New Zealand

**Stephen Finn**

Ireland

**Marco Fiocchetti**

Italy

**Gary Firestone**

USA

**Eva Fischer-Fodor**

Romania

**Carla Fisher**

USA

**Gabriel Fiszman**

Argentina

**Timothy Fitzgerald**

USA

**James Flanagan**

UK

**Linda Fleisher**

USA

**Ana-Maria Florea**

Germany

**Uta Flucke**

Netherlands

**Marco Folini**

Italy

**Gunnar Folprecht**

Germany

**Liz Forbat**

UK

**Cynthia Forbes**

Canada

**Patrick Forde**

USA

**Lynne Forrest**

UK

**Charles Forscher**

USA

**Rosemary Foster**

USA

**Laura Fouassier**

France

**Rezarta Frakulli**

Italy

**Tindara Franchina**

Italy

**Renato Franco**

Italy

**Dale Frank**

Israel

**Nicolaas Franken**

Netherlands

**Steve Fraser**

Australia

**Sarah Freemantle**

USA

**Daniela Frezzetti**

Italy

**Hermann Frieboes**

USA

**Daniel Frigo**

USA

**Patrizia Froesch**

Switzerland

**Marios Froudarakis**

Greece

**Martine Frouws**

Netherlands

**John Fruehauf**

USA

**Michael Frumovitz**

USA

**Liwu Fu**

China

**Bruno Fuchs**

Switzerland

**Enrique Fuentes-Mattei**

USA

**Alexander Fuhrmann**

USA

**Mayumi Fujita**

USA

**Yutaka Fujiwara**

Japan

**Mikihiro Fujiya**

Japan

**Minoru Fukuchi**

Japan

**Valerio Fulci**

Italy

**Stefano Fumagalli**

Italy

**Fiona Furlong**

UK

**Junji Furuse**

Japan

**Mayuko Furuta**

Japan

**Hideki Furuya**

USA

**Manabu Futamura**

Japan

**Anthony Fyles**

Canada

**Shahinaz Gadalla**

USA

**Shiva Keshava Gaddam B**

USA

**Alain-Pierre Gadeau**

France

**Gianluca Gaidano**

Italy

**Nadine T. Gaisa**

Germany

**Umberto Galderisi**

Italy

**Miguel Gallego**

Spain

**Gary Edward Gallick**

USA

**Stefania Gallucci**

USA

**Antoine Galmiche**

France

**Daniel Galvao**

Australia

**Roberto Gambari**

Italy

**Ratish Gambhira**

USA

**Ana Gamero**

USA

**Jamie Bakkum Gamez**

USA

**Simon Peter Gampenrieder**

Austria

**Paolo Gandellini**

USA

**Ruchika Gangwar**

USA

**Ai Gao**

China

**Enrico Garattini**

Italy

**Diego Garcia**

UK

**Javier García Castro**

Spain

**José Manuel García-Heredia**

Spain

**Manoj Garg**

Singapore

**Manuela Gariboldi**

Italy

**Barbara Garmy-Susini**

France

**Aldo Garozzo**

Italy

**Ignacio Garrido-Laguna**

USA

**Dale Garsed**

Australia

**Andrei Gartel**

USA

**James Garvin**

USA

**Mary A. Garza**

USA

**Diana Gaspar**

Portugal

**Kathy Gately**

Ireland

**Laura Gatti**

Italy

**Giovanni Gaudino**

USA

**Julie Gavard**

France

**Patrizia Gazzerro**

Italy

**Lianying Ge**

China

**Ricardo Gehrau**

USA

**Mathias Gehrmann**

Germany

**Alessandra Gennari**

Italy

**Petra Georg**

Austria

**George Georgakis**

USA

**Charles George**

Australia

**Christopher Gerner**

Austria

**Marco Gessi**

Germany

**Olivier Gevaert**

USA

**Rajendra Gharbaran**

USA

**Rahel Ghebre**

USA

**Djamel Ghebriou**

France

**Fabio Ghezzi**

Italy

**Paramita Ghosh**

USA

**Sarbani Ghosh Laskar**

India

**Bruce Giantonio**

USA

**Spencer Gibson**

Canada

**Carolina Gigek**

Brazil

**Joan Gil**

Spain

**Duncan Gilbert**

UK

**David Gilham**

UK

**Sharlene Gill**

Canada

**Theresa W. Gillespie**

USA

**John Gilmer**

Ireland

**Hannah Gilmore**

USA

**Elizabeth Ginsburg**

USA

**Rosaria Giordano**

Italy

**Paolo Giorgi Rossi**

Italy

**David Giovannucci**

USA

**Albert Girotti**

USA

**Francesca Maria Giugliano**

Italy

**Simona Glasberg**

Israel

**Michael Glogauer**

Canada

**Tony Godfrey**

USA

**Vladimir Gogvadze**

Czech Republic

**Gary Goldberg**

USA

**David Goldgar**

USA

**Vita Golubovskaya**

USA

**Catarina Gomes**

Portugal

**Jorge Gomez**

USA

**Pedro Goncalves**

France

**Binsheng Gong**

USA

**Céline Gongora**

France

**Vianey Gonzalez Villasana**

Mexico

**Jerry Goodisman**

USA

**Oscar Goodman**

USA

**Satish Gopal**

Malawi

**Priya Gopalan**

USA

**Chandra Goparaju**

USA

**John Gordan**

USA

**Jesse Gore**

USA

**Jessica Gorman**

USA

**Kylie Louise Gorringe**

Australia

**Rei Goto**

Japan

**Martin Gotthardt**

Netherlands

**Jonathan Gottlieb**

USA

**Raghavendra Gowda**

USA

**Dorthe Grabau**

Sweden

**Anna Grabowska**

UK

**Marco Grados**

USA

**Paola Grandi**

USA

**Cristina Grange**

Italy

**Frederic Grannis**

USA

**Claudia Gravekamp**

USA

**Giovanni Gravina**

Italy

**Richard Gray**

USA

**Jeffrey Green**

USA

**Edward Greenfield**

USA

**Marcel Greuter**

Netherlands

**Richard Griffey**

USA

**Thomas Griffith**

USA

**Filippo Grillo-Ruggieri**

Italy

**Raymon Grogan**

USA

**Christian Grohe**

Germany

**Bjørn H. Grønberg**

Norway

**Jonathan Gross**

USA

**Ana Rita Grosso**

Portugal

**Alejandro Gru**

USA

**Viktor Grünwald**

Germany

**James Grutsch**

USA

**Vitalina Gryshkova**

Belgium

**Xiaoxiang Guan**

China

**Elizabeth Guancial**

USA

**Dominique Guenot**

France

**Marlon Guerrero**

USA

**Rafael Guerrero-Preston**

USA

**Stefano Guerriero**

Italy

**Udayan Guha**

USA

**Parry Guilford**

New Zealand

**Julie Guillermet-Guibert**

France

**Silvia Guil-Luna**

Spain

**Maha Guindi**

USA

**Zhang Guiyu**

China

**Sakir Humayun Gultekin**

USA

**Orlando Guntinas-Lichius**

Germany

**Saketh Guntupalli**

USA

**Huan Guo**

China

**Shikha Gupta**

India

**Jason Gurney**

New Zealand

**Mayda Gursel**

Turkey

**Ana Gutierrez-Fernandez**

Spain

**Mike Guzman**

India

**Balazs Györffy**

Hungary

**Martin Haag**

Germany

**Amy Haberman**

USA

**Mehryar Habibi Roudkenar**

Iran

**Mouhammed Amir Habra**

USA

**Peiman Haddad**

Iran

**Hany Hafiz**

USA

**A. Haggerty**

USA

**Christel Häggström**

Sweden

**Jörg Haier**

Germany

**Russell Hales**

USA

**John Haley**

USA

**Alix Hall**

Australia

**Florian Haller**

Germany

**Oleh Haluszka**

USA

**Yasuo Hamamoto**

Japan

**Chisato Hamashima**

Japan

**Anne Hamburger**

USA

**Henning Hamm**

Germany

**Ester Hammond**

UK

**Wenling Han**

China

**Andrew Hanby**

UK

**Swei Sunny Hann**

China

**Chris Hanrahan**

USA

**Ross Hansen**

Australia

**Jorg Hanze**

Germany

**Anja Harder**

Germany

**Jessica Harding**

Australia

**Paul Harkey**

USA

**Wayne Harris**

USA

**Jaana Hartikainen**

Finland

**Ryan Hartmaier**

USA

**Sheri Hartman**

USA

**Sayed Hashemi**

Netherlands

**Onn Hashim**

Malaysia

**Hazem Hassan**

USA

**Maria Hatziapostolou**

USA

**Matthias Hautmann**

Germany

**Rachel Hazan**

USA

**Rong-Rong He**

China

**Wilma Heemsbergen**

Netherlands

**Christopher Heery**

USA

**Balazs Hegedus**

Austria

**Monika Hegi**

Switzerland

**David Heigener**

Germany

**Mirjam Heinen**

Ireland

**Malgorzata Heinrich**

UK

**Klaus Heißner**

Germany

**Ellen Heitzer**

Austria

**Åslaug Helland**

Norway

**Jozien Helleman**

Netherlands

**Eva Hellmén**

Sweden

**Eric Henderson**

USA

**Christoph Henkenberens**

Germany

**James Hennessey**

USA

**Marie Henriksson**

Sweden

**Rui Henrique**

Portugal

**Kevin Henry**

USA

**Anna Herman-Antosiewicz**

Poland

**Mario Hermsen**

Spain

**Luis Hernandez**

Spain

**David Hernandez Gonzalo**

USA

**Hector Hernandez-Vargas**

France

**Jolyn Hersch**

Australia

**Pamela Hershberger**

USA

**Shawn Hervey-Jumper**

USA

**Lisa Hess**

USA

**Bart Hesselink**

Netherlands

**Marcin Hetnal**

Poland

**Martin Heur**

USA

**Paul Hewson**

UK

**Ryan Hickey**

USA

**Katalin Hideghéty**

Hungary

**Thomas Hielscher**

Germany

**Elizabeth Hill**

UK

**Gunnar Hillerdal**

Sweden

**Sebastian Hinz**

Germany

**Matthias Hipp**

Germany

**Yoshihiko Hirohashi**

Japan

**Megan Hitchins**

USA

**Taro Hitosugi**

USA

**Gwo Fuang Ho**

Malaysia

**David Hoaglin**

USA

**Robert Hobbs**

USA

**Howard Hochster**

USA

**Steven Hochwald**

USA

**Markus Hoffermann**

Austria

**Robert Hoffman**

USA

**Paul Hofman**

France

**Martin Hoglund**

Sweden

**Jörg Hoheisel**

Germany

**Bryan Holcomb**

USA

**Randall Holcombe**

USA

**Antoine Hollebecque**

France

**Marcus Hollenbach**

Germany

**Peter Hollenhorst**

USA

**Linda Holmquist Mengelbier**

Sweden

**Cheryl Holt**

USA

**Klaus Holzmann**

Austria

**Sabine Hombach-Klonisch**

Canada

**Akihiro Homma**

Japan

**Jie Hong**

China

**Satoshi Honjo**

Japan

**Elizabeth Hopper-Borge**

USA

**Muhammad Hoque**

South Africa

**Marcus Horstmann**

Germany

**Kent Hoskins**

USA

**Naoko Hosono**

Japan

**Yu-Chih Hou**

Taiwan

**Serena C. Houghton**

USA

**Sacha Howell**

UK

**Lynne Howells**

UK

**Philip Howles**

USA

**Michael Hsiao**

Taiwan

**Yi-Hsien Hsieh**

Taiwan

**Po-Kuei Hsu**

Taiwan

**Dong Gui Hu**

Australia

**Yunpeng Hua**

China

**Changzhi Huang**

China

**Michael Hubalek**

Austria

**Christian Hübbers**

Germany

**Megan Huchko**

USA

**John Hughes**

UK

**Winston Huh**

USA

**Luo Huiyan**

China

**Amanda Hummon**

USA

**Matthew Hunt**

USA

**Keith Hunter**

UK

**Ted Hupp**

UK

**Robert Hurst**

USA

**Juliette Hussey**

Ireland

**Dietmar Werner Hutmacher**

Australia

**Bonnie Hylander**

USA

**Alena Hyrslova-Vaculova**

Czech Republic

**Roberto Iacovelli**

Italy

**Takafumi Ichida**

Japan

**Solachuddin J. A. Ichwan**

Malaysia

**Kenneth Iczkowski**

USA

**Carolina Ilkow**

Canada

**Mohammad Ilyas**

UK

**Haejin In**

USA

**Evan Ingley**

Australia

**Wendy Ingman**

Australia

**Johanna Inhestern**

Germany

**Pasquale Innominato**

France

**Kaire Innos**

Estonia

**Hidenori Inohara**

Japan

**Takeshi Inoue**

Japan

**Monica Iorfida**

Italy

**Hassan Iqbal**

Pakistan

**Rosalyn Irby**

USA

**Will Irving**

UK

**Nobuhisa Ishikawa**

Japan

**Ciro Isidoro**

Italy

**Yukako Ito**

Japan

**Orit Itzhaki**

Israel

**Mircea Ivan**

USA

**Motoki Iwasaki**

Japan

**Evgeny Izumchenko**

USA

**Julie Izzo**

USA

**Sonia Jacinto**

Philippines

**Maria Jackson**

Jamaica

**Rojymon Jacob**

USA

**Ramune Jacobsen**

Denmark

**Geraldine Jacobson**

USA

**Richard Jäger**

Germany

**Preetesh Jain**

USA

**Jens Jakob**

Germany

**Venkatakrishna Jala**

USA

**Emilia Jalil**

Brazil

**Michael James**

USA

**Mark Jameson**

USA

**Elzbieta Janda**

Italy

**Peter Janes**

Australia

**Feliksas Jankevicius**

Lithuania

**Maurice Jansen**

Netherlands

**Joseph Janssen**

Netherlands

**Tavan Janvilisri**

Thailand

**Gul Javid**

India

**Seyed Mohammad Jazayeri**

Iran

**Krystian Jazdzewski**

Poland

**Pujol Jean Louis**

France

**Patcharee Jearanaikoon**

Thailand

**Wojciech Jelski**

Poland

**Brendan Jenkins**

Australia

**Stephen Jennings**

USA

**Furlanetto Jenny**

Germany

**Jørgen Bjerggaard Jensen**

Denmark

**Florian Jentzmik**

Germany

**Christine Jeoffrion**

France

**Sangchoon Jeon**

USA

**Joon Jeong**

South Korea

**Fredrik Jerhammar**

Sweden

**Mats Jerkeman**

Sweden

**Carmen Jeronimo**

Portugal

**Udo Jeschke**

Germany

**Rinath Jeselsohn**

USA

**Mahsa Jessri**

Canada

**Bruno Jham**

USA

**Chunyan Ji**

China

**Rui-Peng Jia**

China

**Wei Jiang**

China

**Young-Joo Jin**

South Korea

**Sonali Jindal**

USA

**Keavin Jing**

South Korea

**Kirsten Jochumsen**

Denmark

**Mattias Johansson**

France

**James Johnson**

Canada

**Donna Johnston**

Canada

**Kevin Jones**

USA

**Simon Joosse**

Germany

**Susan Jordan**

Australia

**Katja Jordanova**

Netherlands

**Marit Eika Jørgenen**

Denmark

**Kurian Jones Joseph**

Canada

**Monika Joshi**

USA

**Philipp Jost**

Germany

**Jingfang Ju**

USA

**Angeles Juarranz**

Spain

**Louise Judd**

Australia

**Gabor Juhasz**

Hungary

**Alain Jung**

France

**Kerstin Junker**

Germany

**Elona Juozaityte**

Lithuania

**Paul Jurasz**

Canada

**Vladi Juric**

USA

**Vindi Jurinovic**

Germany

**Guillermo Juvenal**

Argentina

**Pinar Kadioglu**

Turkey

**Daniel Kaemmerer**

Germany

**Larry Kaiser**

USA

**John Kalbfleisch**

USA

**Vijaykumar Kale**

USA

**Pawel Kalinski**

USA

**Konstantine Kalogeras**

Greece

**Holger Kalthoff**

Germany

**Arjun Kalvala**

USA

**Takao Kamai**

Japan

**Arif Kamal**

USA

**Balu Kamaraj**

India

**Izumi Kamiya**

Japan

**Yogita Kanan**

USA

**Rama Krishna Kancha**

India

**Raju Kandimalla**

Netherlands

**Mustapha Kandouz**

USA

**Eleanor Kane**

UK

**Kazunori Kanehira**

USA

**Peggy Kanellou**

Greece

**Stephen Kang**

USA

**Paivi Kannisto**

Sweden

**Maya Kansara**

Australia

**Sung-Shuo Kao**

Taiwan

**Akhil Kapoor**

India

**Niki Karachaliou**

Spain

**George Karagkounis**

USA

**Cansu Karakas**

USA

**Sherif Karam**

United Arab Emirates

**Aleksandar Karamarkovic**

Serbia

**Deepkamal Karelia**

USA

**Peeter Karihtala**

Finland

**Surajit Karmakar**

India

**Thomas Karn**

Germany

**Mehdi Karoui**

France

**Igor Karp**

Canada

**Georg Karpel-Massler**

USA

**Adam R. Karpf**

USA

**Michael Karsy**

USA

**Barbara Karten**

Canada

**Daniel Kaschek**

Germany

**Andrea Kasinski**

USA

**Hidetaka Katabuchi**

Japan

**Hideki Katagiri**

Japan

**Kota Katanoda**

Japan

**Maria Katapodi**

Switzerland

**Tejinder Kataria**

India

**Sanjay Katiyar**

USA

**Takamitsu Kato**

USA

**Masaru Katoh**

Japan

**Aviva Katz**

USA

**Pravin Kaumaya**

USA

**Sukhwinder Kaur**

USA

**Andrew Kaz**

USA

**Youqiang Ke**

UK

**Bhumsuk Keam**

South Korea

**Adam Keeton**

USA

**Benjamin Kefas**

USA

**Fergal Kelleher**

Ireland

**Brad Keller**

USA

**Stefan Kempa**

Germany

**Hirotsugu Kenmotsu**

Japan

**Catherine Kennedy**

Australia

**Patrick Kenney**

USA

**Deanna Kepka**

USA

**Shiva Keshava**

USA

**Ken Kesler**

USA

**Nana Keum**

USA

**Khandan Keyomarsi**

USA

**Salma Khan**

USA

**Sok Khoo**

USA

**Zhamak Khorgami**

USA

**Omid Khorram**

USA

**Shahram Khosravi**

Canada

**Michel Khouri**

USA

**Darren Kies**

USA

**Tobias Kiesslich**

Austria

**Jens Folke Kiilgaard**

Denmark

**Misato Kikuchi**

Japan

**Robert Kilgour**

Canada

**John Kiluk**

USA

**Hirshfield Kim**

USA

**Ingorn Kimkong**

Thailand

**Takahiro Kimura**

Japan

**Tamila Kindwall-Keller**

USA

**Rhonda Kineman**

USA

**Karl Kingsley**

USA

**Tomas Kirchhoff**

USA

**Jolle Kirpensteijn**

Netherlands

**Lawrence Kirschner**

USA

**Cliona Kirwan**

UK

**Chagriya Kitiyakara**

Thailand

**Antti Kivelä**

Finland

**Marc Kiviniemi**

USA

**Kristina Kjaerheim**

Norway

**Ann Klassen**

USA

**Christoph Klein**

Germany

**Samuel Klempner**

USA

**Gregory Kline**

Canada

**Merieme Klobocista**

USA

**Helmut Klocker**

Austria

**Matthias Kloor**

Germany

**Stefanie Klug**

Germany

**Jeffrey Knauf**

USA

**Ilka Knippertz**

Germany

**Klaus-Peter Knoeloch**

Germany

**Lucia Knopfová**

Czech Republic

**Ji Hyun Ko**

Canada

**Lindsay Kobayashi**

UK

**Maheedhar Kodali**

USA

**Jyoti Kode**

India

**Corinna Koebnick**

USA

**Bruno Christian Koehler**

Germany

**Christian Koelsche**

Germany

**Viktor H Koelzer**

Switzerland

**Fumitaka Koga**

Japan

**Donat Kögel**

Germany

**Manolis Kogevinas**

Spain

**Takashi Kojima**

Japan

**Klass Kok**

Netherlands

**E. Anders Kolb**

USA

**Matthias Kolberg**

Norway

**Jill Kolesar**

USA

**Frank Thomas Kölligs**

Germany

**Yoshihiro Komohara**

Japan

**Joseph Cherng Huei Kong**

Australia

**Gordana Konjevic**

Serbia

**Christos Kontos**

Greece

**Dana-Lynn Koomoa**

USA

**Hon Wai Koon**

USA

**Marianne Koritzinsky**

Canada

**Steven Kornblau**

USA

**Gabriela Kornek**

Austria

**Alexander Kornienko**

USA

**Nobuyoshi Kosaka**

Japan

**Masafumi Koshiyama**

Japan

**Ioannis Kotsianidis**

Greece

**George Koutsodontis**

Greece

**Zaklina Kovacevic**

Australia

**Artur Kowalik**

Poland

**Sergei Kozlov**

Australia

**Jamie Kramer**

Canada

**Matthew Krebs**

UK

**Friedrich Kreth**

Germany

**Smriti Krishna**

Australia

**Moorthy Krishnan**

USA

**Lasse Sommer Kristensen**

Denmark

**George Kroumpouzos**

USA

**Marija Krstic-Demonacos**

UK

**Sebastian Krug**

Germany

**Rosemarie Krupar**

Germany

**Rui Kuang**

USA

**Shuji Kubo**

Japan

**Alexander Kudinov**

USA

**Thomas Kuhnt**

Germany

**Roland Kuiper**

Netherlands

**Seval Kul**

Turkey

**Alok Kumar**

USA

**Chandan Kumar-Sinha**

USA

**Regalla Kumarswamy**

Germany

**Gopal Kundu**

India

**John Kuo**

USA

**Charlotte Kuperwasser**

USA

**Peter J.K. Kuppen**

Netherlands

**Balamurugan Kuppusamy**

USA

**Hidekazu Kuramochi**

Japan

**Junya Kuroda**

Japan

**Kazuhiko Kurozumi**

Japan

**Hiroyuki Kuwano**

Japan

**Young Kwok**

USA

**Ava Kwong**

Hong Kong

**Natasha Kyprianou**

USA

**Kyriacos Kyriacou**

Cyprus

**Marie-Christine Kyrtsonis**

Greece

**James Labelle**

USA

**Boleslaw Lach**

Canada

**Stephen Lagana**

USA

**Dongmei Lai**

China

**Ite A. Laird-Offringa**

USA

**Ram Lakhan**

USA

**Ashish Lal**

USA

**Lloyd Lam**

USA

**Diane Lambert**

France

**Nadia Lampiasi**

Italy

**Andrea Lampis**

UK

**Gene Landon**

USA

**Darius Lane**

Australia

**Joshua Lang**

USA

**Rupert Langer**

Switzerland

**Scott Langevin**

USA

**Andrea Lania**

Italy

**Malinee Laopaiboon**

Thailand

**Constantin Lapa**

Germany

**Alexey Larionov**

UK

**Ulrik Lassen**

Denmark

**Peter Lauf**

USA

**Benoit Laurent**

USA

**André Lavorato-Rocha**

Brazil

**Alfons Lawen**

Australia

**Ben Lawrence**

New Zealand

**Richard Le Leu**

Australia

**Jayanthi Lea**

USA

**Amy E. Leader**

USA

**Tomas Leanderson**

Sweden

**Vijittra Leardkamolkarn**

Thailand

**Patrick Leavey**

USA

**Richard Lebaron**

USA

**Bonita Lee**

Canada

**Simon Leedham**

UK

**Alan Lefor**

Japan

**Markos Leggas**

USA

**Sophie Lehn**

Sweden

**Catherine Lejeune**

France

**Jochen Lennerz**

USA

**Charles Leonard**

USA

**Lorenzo Leoncini**

Italy

**Andreia Leopoldino**

Brazil

**Adam Lerner**

USA

**Gregory Lesinski**

USA

**Joanne Lester**

USA

**Yi-Shing Leu**

Taiwan

**Samuel Leung**

Canada

**Charles Levea**

USA

**Francis Levi**

France

**Ephrat Levy-Lahad**

Israel

**Joan Lewis-Wambi**

USA

**Jeffrey Leyton**

Canada

**Chien-Feng Li**

Taiwan

**Ting-Bo Liang**

China

**Evi Lianidou**

Greece

**Massimo Libra**

Italy

**Jonathan Licht**

USA

**Sandra Liekens**

Belgium

**Astrid Lièvre**

France

**Michael Lilly**

USA

**Anita Lim**

UK

**Zhen Lin**

Australia

**Amy Linabery**

USA

**Isabel Linares**

Spain

**Mårten Lindén**

Sweden

**Linda Sofie Lindström**

Sweden

**Jun Ling**

USA

**Alexander Link**

Germany

**Michael Linnebacher**

Germany

**Esther Lips**

Netherlands

**Steven Lipshultz**

USA

**Kriengsak Lirdprapamongkol**

Thailand

**Lukasz Liszka**

Poland

**Virginia Litle**

USA

**Laurie Littlepage**

USA

**Stanley Liu**

Canada

**Sigita Liutkauskiene**

Lithuania

**Xavier Llor**

USA

**Ana Lluch**

Spain

**Hui-Wen Lo**

USA

**Arturo Loaiza-Bonilla**

USA

**Joseph Locker**

USA

**Trevor Lockett**

Australia

**Filippo Lococo**

Italy

**Loretta Loftus**

USA

**Rasiah Loganantharaj**

USA

**Birgit Lohberger**

Austria

**Sherene Loi**

Australia

**Martijn Lolkema**

Netherlands

**Gwen Lomberk**

USA

**Fulvio Lonardo**

USA

**Priya Londhe**

USA

**Kara Long**

USA

**Daniel Longley**

UK

**Valter Longo**

USA

**Per Lonning**

Norway

**Lenora Loo**

USA

**Alessia Lopergolo**

Italy

**Kristina Lorenz**

Germany

**Ian Lorimer**

Canada

**Attila Lorincz**

UK

**Pei-Jen Lou**

Taiwan

**Maeve Lowery**

USA

**Yuanyuan Lu**

China

**David Lubaroff**

USA

**Claudio Luchini**

Italy

**Heinz Ludwig**

Germany

**Alessandro Lugli**

Switzerland

**Geanncarlo Lugo**

France

**Jennifer Lund**

USA

**Samuel Lundin**

Sweden

**Hong Lok Lung**

Hong Kong

**Sheng-Dean Luo**

Taiwan

**John Lurain**

USA

**Charles Lutz**

USA

**Michael Lux**

Germany

**Herbert Lyerly**

USA

**Craig Lynch**

Australia

**David Ma**

Canada

**Jorma Antero Määttä**

Finland

**Teresa Macarulla**

Spain

**Antonio Macciò**

Italy

**Muzafar Macha**

USA

**Robert MacInnis**

Australia

**Michael Mackenzie**

USA

**Heath Mackley**

USA

**Roderick MacLeod**

Australia

**Jonna Madsen**

Denmark

**Yoshihiko Maehara**

Japan

**Andrea Mafficini**

Italy

**Mark Jesus Magbanua**

USA

**Luca Magnani**

UK

**Jamal Mahajna**

Israel

**Christopher Maher**

USA

**Anuj Mahindra**

USA

**Habibollah Mahmoodzadeh**

Iran

**Hai-Qiang Mai**

China

**Flavio Maina**

France

**Sankar Maity**

USA

**Nk Mak**

Hong Kong

**Talia Malagon**

Canada

**Pasqualino Malandrino**

Italy

**Núria Malats**

Spain

**Carl Malchoff**

USA

**Jitka Malcikova**

Czech Republic

**Ashim Malhotra**

USA

**George Malietzis**

UK

**Judith Malmgren**

USA

**Gabriel Malouf**

France

**Marcelo Mamede**

Brazil

**Cyril Mamotte**

Australia

**Parmeet Manchanda**

USA

**Julien Mancini**

France

**Raju Kumar Mandal**

India

**Stéphane Mandard**

France

**Haresh Mani**

USA

**Lorella Maniscalco**

Italy

**Bruce Mann**

Australia

**Lorenzo Mannelli**

USA

**Ferdinando Mannello**

Italy

**Aaron Mansfield**

USA

**Ulrich Mansmann**

Germany

**Zhiyong Mao**

China

**Sergio Marchini**

Italy

**Caterina Marchiò**

Italy

**Johanna Maree**

South Africa

**Vitaly Margulis**

USA

**Catalin Marian**

Romania

**Isabelle Maridonneau-Parini**

France

**Laure Marignol**

Ireland

**Patricia Marino**

France

**Michael Luke Marinovich**

Australia

**Talar Markossian**

USA

**Pablo Maroto**

Spain

**Vincenzo Marotta**

Italy

**Rebecca Marquez**

USA

**Iván Márquez-Rodas**

Spain

**Jean Marshall**

Canada

**Finian Martin**

Ireland

**Maria L. Martinez Chantar**

Spain

**Massimo Mascolo**

Italy

**Antonio Masi**

USA

**Khalid Matalka**

Jordan

**Larry Matherly**

USA

**Sachiko Matsuhashi**

Japan

**Kazumasa Matsumoto**

Japan

**Yoshiaki Matsumura**

Japan

**Koji Matsuo**

USA

**Junko Matsuzaki**

USA

**Jaime Matta**

USA

**Matthias Matter**

Switzerland

**Vance Matthews**

Australia

**Aras Mattis**

USA

**Ines Mattos**

Brazil

**Vsevolod Matveev**

Russian Federation

**Fekicity Eb May**

UK

**Erik Mayer**

UK

**Maria Maynard**

UK

**Olive Mbah**

USA

**Sam Mbulaiteye**

USA

**Stephanie McArdle**

UK

**John McAuliffe**

USA

**Matthew Nicholson McCall**

USA

**Georgia McCann**

USA

**Nina McCarthy**

Australia

**Joseph McCarty**

USA

**Michael McDevitt**

USA

**Jasmine McDonald**

USA

**Cedrek McFadden**

USA

**Ronald McGarry**

USA

**Victoria McGilligan**

UK

**Susan McGovern**

USA

**Jane McHowat**

USA

**Alan McIntyre**

UK

**Mark McKeage**

New Zealand

**Sarah McLaughlin**

USA

**Philip McLoone**

UK

**Tara McMorrow**

Ireland

**Kristen McNamara**

USA

**Stephen McPherson**

Australia

**Imen Medimegh**

Tunisia

**Aurora Medina-Sanson**

Mexico

**Michal Mego**

Slovakia

**Gabor Mehes**

Hungary

**Ranee Mehra**

USA

**Sumita Mehta**

India

**Davide Melisi**

Italy

**Anders Mellemgaard**

Denmark

**Inga Maria Melzer**

Germany

**William Mendenhall**

USA

**Le Meng**

USA

**Douglas Scott Merrell**

USA

**Neil Merrett**

Australia

**Joachim Mertens**

Switzerland

**Linda Metheny-Barlow**

USA

**Ayman Metwally**

Egypt

**Roman Mezencev**

USA

**Jörg Mezger**

Germany

**Zhiyong Mi**

USA

**Aisha Miah**

UK

**Ruoyu Miao**

USA

**J.S. Michaelson**

USA

**Loren Michel**

USA

**Pellegrino Michele**

Italy

**David Midthun**

USA

**Antimo Migliaccio**

Italy

**Toshinari Mikami**

Japan

**Atsushi Miki**

Japan

**Wolfgang Mikulits**

Austria

**Michael Milano**

USA

**Karin Milde-Langosch**

Germany

**Anne Miles**

UK

**Emma Miller**

Australia

**Nathaniel Milton**

UK

**Daniel Mina**

Canada

**Seigo Minami**

Japan

**Ryogo Minamimoto**

Japan

**Janos Minarovits**

Hungary

**Christoph Minichsdorfer**

Austria

**Maria Mione**

Germany

**Kiyoshi Misawa**

Japan

**Dhruva Mishra**

USA

**Paul Mitchell**

Australia

**Jeffrey Mito**

USA

**Siddhartha Mitra**

USA

**Naoto Miura**

Japan

**Makito Miyake**

Japan

**David Miyamoto**

USA

**Minoru Miyashita**

Japan

**Yasuyoshi Miyata**

Japan

**Shinjiro Mizuguchi**

Japan

**Yoichi Mizukami**

Japan

**Eishiro Mizukoshi**

Japan

**Yoshiyuki Mizushina**

Japan

**Markus Möbs**

Germany

**Dominik Modest**

Germany

**Naomi Modeste**

USA

**Helmout Modjtahedi**

UK

**Michele Modugno**

Italy

**Markus Moehler**

Germany

**Siver Andreas Moestue**

Norway

**Khalid Mohamedali**

USA

**Ramzi Mohammad**

USA

**Anant Mohan**

India

**Yejin Mok**

South Korea

**Nadia Mokhtar**

Egypt

**Manuel Molina**

USA

**Solange Moll**

Switzerland

**Kevin Monahan**

UK

**Chiara Mondello**

Italy

**Alison Mondul**

USA

**Cristina Montagna**

USA

**Maria Theresa Montales**

USA

**Monica Montano**

USA

**Robson Monteiro**

Brazil

**Michael Montemurro**

Switzerland

**Corey Montgomery**

USA

**Jennifer Moodley**

South Africa

**Sung-Kwon Moon**

South Korea

**Harvey Moore**

USA

**Patricia Moorman**

USA

**Christudas Morais**

Australia

**Martin Mørck Mortensen**

Denmark

**James Morden**

UK

**Violaine Moreau**

France

**Gema Moreno-Bueno**

Spain

**Peter Morfeld**

Germany

**Alicia Morgans**

USA

**Daniel Morgensztern**

USA

**Floriana Morgillo**

Italy

**Branden Moriarity**

USA

**Eiichi Morii**

Japan

**Yuji Morine**

Japan

**Toshikazu Moriwaki**

Japan

**Hans Morreau**

Netherlands

**Mohamed Moustafa**

Lebanon

**Ray Moynihan**

Australia

**Michael Muders**

Germany

**Jenna Mueller**

USA

**Anne Muesch**

USA

**Dirk Mügge**

Germany

**Malini Mukherjee**

USA

**William Muller**

USA

**Ashok Muniappan**

USA

**Montse Muñoz**

Spain

**Sandra Muñoz-Galván**

Spain

**Alastair Munro**

UK

**Masanao Murakami**

Japan

**Neil Murphy**

UK

**Nevin Murray**

Canada

**D.J. Murry**

USA

**Charles Murtauh**

USA

**Paari Murugan**

USA

**Lucia Anna Muscarella**

Jordan

**Karthikeyan Muthusamy**

India

**Ravikumar Muthuswamy**

USA

**Anthony Mutsaers**

Canada

**Karthikeyan Mythreye**

USA

**Shunji Nagai**

USA

**Pratik Nagaria**

USA

**Kelly Nagasawa**

USA

**Gabriele Nagel**

Germany

**Seema Nagpal**

USA

**Shigeki Nakagawa**

USA

**Yasushi Nakai**

Japan

**Tatsuya Nakamura**

USA

**Tomio Nakayama**

Japan

**Antonella Naldini**

Italy

**Nisana Namwat**

Thailand

**Hongmei Nan**

USA

**Meera Nanjundan**

USA

**Arlene Naranjo**

USA

**Gopeshwar Narayan**

India

**Maja Narlik-Grassow**

Germany

**Steven Narod**

Canada

**Aziza Nassar**

USA

**Mohd Nasser**

USA

**Arutselvan Natarajan**

USA

**David Nathanson**

USA

**Michael Naughton**

USA

**Maria Cristina Navas**

Colombia

**Steffan Nawrocki**

USA

**Sarah Nechuta**

USA

**Deborah Neklason**

USA

**Delia Nelson**

Australia

**Szilard Nemes**

Sweden

**Paolo Netti**

Italy

**Patrick Neven**

Belgium

**Siok Bian Ng**

Singapore

**Min En Nga**

Singapore

**Tudung Nguyen**

USA

**Finn Cilius Nielsen**

Denmark

**Taina Nieminen**

Finland

**Giuseppe Nigri**

Italy

**Naoki Niikura**

Japan

**Anna Nikonova**

USA

**Donna Nile**

UK

**Katharina Nimptsch**

Germany

**Hiroshi Nishida**

Japan

**Reiko Nishihara**

USA

**Benjamin Nisman**

Israel

**Dorothea Nitsch**

UK

**Takeaki Nitto**

Japan

**Andrew Nixon**

USA

**Sisse Helle Njor**

Denmark

**Rosa Noguera**

Spain

**Sung Hoon Noh**

South Korea

**Masanori Nojima**

Japan

**Vikki Noonan**

USA

**Helena Nordenstedt**

Sweden

**Daniel Normolle**

USA

**Sara Notaro**

Austria

**George Notas**

Greece

**Mehdi Nouraie**

USA

**Maciej Nowacki**

Poland

**Susan Nozell**

USA

**Jeremie Nsengimana**

UK

**Lars Ny**

Sweden

**Mukesh Nyati**

USA

**Mari Nygård**

Norway

**Peter Odermatt**

Switzerland

**Olumuyiwa Odusanya**

Nigeria

**Tetsuya Ogawa**

Japan

**Steven Ogbourne**

Australia

**Shuji Ogino**

USA

**Olorunseun Ogunwobi**

USA

**Shun-Ichiro Ogura**

Japan

**Sei-Rynag Oh**

South Korea

**Kazumasa Ohashi**

Japan

**Koji Ohnishi**

Japan

**Nitin Ohri**

USA

**Junko Ohyashiki**

Japan

**Seiichi Okabe**

Japan

**Yusuke Okuma**

Japan

**Matthew Old**

USA

**Leticia Oliveira-Ferrer**

Germany

**Trudy Oliver**

USA

**Magali Olivier**

France

**Robert Olson**

Canada

**Adam Olszewski**

USA

**Elisa Oltra**

Spain

**Mairin O’Mahony**

Ireland

**David O Malley**

USA

**Brigitta Omazic**

Sweden

**Jason Ong**

Australia

**Ana Ortega Eslava**

Spain

**Barbara A. Osborne**

USA

**Gemma Osca**

Spain

**Anton Oseledchyk**

Germany

**Feras Oskan**

Germany

**Mohammed Osman**

Egypt

**Piet Ost**

Belgium

**Christian Ostheimer**

Germany

**Julie Ostrander**

USA

**Wenbin Ou**

China

**Tobias Overbeck**

Germany

**Thomas Ow**

USA

**Taofeek Owonikoko**

USA

**Enver Ozer**

Venezuela

**Michael Paech**

Australia

**Katiucia Paiva**

Brazil

**Marina Pajic**

Australia

**Smarajit Pal**

India

**Emanuela Palmerini**

Italy

**Giovannella Palmieri**

Italy

**Moritz Palmowski**

Germany

**Grazia Palomba**

Italy

**Li Pan**

China

**Massimo Pancione**

Italy

**Aditya K. Panda**

India

**Mala Pande**

USA

**Manoj Pandey**

USA

**Xiufeng Pang**

China

**Benedict Panizza**

Australia

**Cédric Michael Panje**

Germany

**Gaetano Paone**

Italy

**Eirini Papapetrou**

USA

**Emmanouil Pappou**

USA

**Luis A. Pardo**

Germany

**Alexander Parikh**

USA

**Paul Park**

Canada

**Paola Parrella**

Italy

**Toshima Parris**

Sweden

**Alan Parrish**

USA

**Venkata Parsa**

USA

**Wendy Parulekar**

Canada

**Jenny Parvani**

USA

**Valeria Pascale**

Italy

**Roberto Passera**

Italy

**Sandra Pastorino**

USA

**Manuel Alfonso Patarroyo**

Colombia

**Sapna Patel**

USA

**Ian Paterson**

Malaysia

**Chandramani Pathak**

India

**Deepa Patil**

USA

**Santosh Patnaik**

USA

**Ruth Patterson**

USA

**Catherine Paul**

France

**Kajsa Paulsson**

Sweden

**Sandra Pavey**

Australia

**Samantha Payne**

UK

**Inge Pedersen**

Denmark

**Naveen Pemmaraju**

USA

**Christopher Penfold**

UK

**Guang Peng**

USA

**Livius Penter**

Germany

**Zsófia Pénzváltó**

Hungary

**Caterina Peraldo-Neia**

Italy

**Paola Perego**

Italy

**Julia Perera Bel**

Germany

**Cesar Perez**

USA

**Ricardo Perez-Tomas**

Spain

**Suraj Peri**

USA

**Claire Perks**

UK

**Francesco Perri**

Italy

**Federica Perrone**

Italy

**Antoinette Perry**

Ireland

**Sujata Persad**

Canada

**Vivekanandan Perumal**

India

**Janette Perz**

Australia

**Peizhong Peter Wang**

Canada

**Paul Peters**

Canada

**Sven Petersen**

Germany

**Luke Peterson**

USA

**Antonello Petrella**

Italy

**James John Petrik**

Canada

**Armen Petrosyan**

USA

**Karl Ulrich Petry**

Germany

**Stefano Petti**

Italy

**P. Terry Phang**

Canada

**Siobhan Phillips**

USA

**Amanda Phipps**

USA

**Melissa Picard**

USA

**Pier Paolo Piccaluga**

Italy

**Maria Carmela Piccirillo**

Italy

**Alain Piche**

Canada

**Joel Picus**

USA

**Antonio Pierini**

Italy

**Marco Pierotti**

Italy

**Pages Pierre-Benoit**

France

**Steffi Pigorsch**

Germany

**Albrecht Piiper**

Germany

**Renate Pilz**

USA

**Iryna Pinchuk**

USA

**Susan M. Pinney**

USA

**Paul Pinsky**

USA

**Miguel A. Piris**

Spain

**Salvatore Piscuoglio**

USA

**Olga Piskareva**

Ireland

**Manop Pithukpakorn**

Thailand

**Dimitris Placantonakis**

USA

**Elizabeth Plimack**

USA

**Marya Plotkin**

Tanzania

**Martin Pölcher**

Germany

**Patsie Polly**

Australia

**Alfonso Pompella**

Italy

**Tanett Pongtheerat**

Thailand

**Sreenivasan Ponnambalam**

UK

**Elizabeth Poole**

USA

**Luca Porcu**

Italy

**Paola Postacchini**

Italy

**David Potter**

USA

**George Poultsides**

USA

**Celine Pourreyron**

UK

**Chandra Prakash Prasad**

Sweden

**Aleix Prat**

Spain

**Selvaraj Pravink**

USA

**Hans Prenen**

Belgium

**Rebecca Previs**

USA

**Ivanka Prichard**

Australia

**Anna Prizment**

USA

**Supannee Promthet**

Thailand

**Jenifer Prosperi**

USA

**Steven Proulx**

Switzerland

**Sandhya Pruth**

USA

**Darko Pucar**

USA

**Trevor Pugh**

Canada

**Shannon Puhalla**

USA

**Joan Anton Puig Butille**

Spain

**Miguel Angel Pujana**

Spain

**Cornelis Punt**

Netherlands

**Chamindie Punyadeera**

Australia

**Tom Purdie**

Canada

**Kristen Purrington**

USA

**Tracy Putoczki**

Australia

**Ramesh Puttalingaiah**

USA

**Roberto Puxeddu**

Italy

**Chris Pyke**

Australia

**Yuliya Pylayeva-Gupta**

USA

**Wei-Xiang Qi**

China

**Wei Qian**

USA

**Ren-Yi Qin**

China

**Hong Qiu**

USA

**Xiujuan Qu**

China

**Luca Quagliata**

Switzerland

**Elena Quaglino**

Italy

**Marcelo Queiroz**

Switzerland

**Victor Quesada**

Spain

**Quincy Quick**

USA

**David Quigley**

USA

**Zaina Qureshi**

USA

**Camilla Qvortrup**

Denmark

**Zaid R. Al-Wahab**

USA

**Bernard Rachet**

UK

**Ishwar Radhakrishnan**

USA

**Ami Radunskaya**

USA

**Carlin Rafie**

USA

**Kanwal Raghav**

USA

**Lisa Rahangdale**

USA

**Nuh Rahbari**

Germany

**Ruman Rahman**

UK

**Priyamvada Rai**

USA

**Komal Raina**

USA

**Madhwa Raj**

USA

**Rajiv Rajani**

USA

**Markus Ralser**

UK

**Ram Mohan Ram Kumar**

Switzerland

**Kunnambath Ramadas**

India

**Anand Ramanathan**

Malaysia

**Pedro Ramirez**

USA

**Theodoros Rampias**

USA

**Kerstin Ramser**

Sweden

**Sophia Ran**

USA

**Aditi Ranade**

USA

**Mark Ranalli**

USA

**Leslie Randall**

USA

**Girolamo Ranieri**

Italy

**Anna Ranta**

New Zealand

**Guglielmina Ranzani**

Italy

**Ganesh Rao**

USA

**Aaron Rapoport**

USA

**Dominique Rash**

USA

**Leon Raskin**

USA

**Andrea Rasola**

Italy

**Pramod Rath**

India

**Appu Rathinavelu**

USA

**Edward Ratovitski**

USA

**Ramandeep Rattan**

USA

**Tilman Rau**

Switzerland

**Paul Rava**

USA

**Manmeet Raval**

USA

**Pritha Ray**

India

**Adam Raymakers**

Canada

**Azad Razack**

UK

**Olga Razorenova**

USA

**Nishitha Reddy**

USA

**Kerryn Reding**

USA

**Françoise Rédini**

France

**Nicholas Redshaw**

UK

**Karen Reed**

UK

**Jeremy Rees**

UK

**Marina Reeves**

Australia

**Mauricio Reginato**

USA

**Glen Reid**

Australia

**Anne Reiman**

UK

**Nickolas Reimer**

USA

**Raquel Reinbolt**

USA

**Anne Reiner**

USA

**Niels Reinmuth**

Germany

**Rui Reis**

Brazil

**Peter Reiser**

USA

**David Reisman**

USA

**Zefang Ren**

China

**Diana Resendez**

Mexico

**Edyta Reszka**

Poland

**Miriam Reuschenbach**

Germany

**Brent Rexer**

USA

**Marc-André Reymond**

Germany

**Hamid Reza Rezvani**

France

**Josep-Maria Ribera**

Spain

**Fulvio Ricceri**

Italy

**Luigi Ricciardiello**

Italy

**Feltbower Richard**

UK

**Debra Richardson**

USA

**Timothy Richmond**

USA

**Assunta Rienzo**

USA

**Piotr Rieske**

Poland

**Jonathan Riess**

USA

**Rebecca Riggins**

USA

**Guido Rindi**

Italy

**April Risinger**

USA

**Michael Risk**

USA

**Karin Roberg**

Sweden

**Jacques Robert**

France

**Ryan Roberts**

USA

**Cleo Robinson**

Australia

**Trude Eid Robsahm**

Norway

**Kara Long Roche**

Japan

**Robert Rodnitzky**

USA

**Kathryna Rodrigues**

Brazil

**Sara Rodriguez-Enriquez**

Mexico

**Kenny A. Rodriguez-Wallberg**

Sweden

**Silvia Rogatto**

Brazil

**Sabine Rohrmann**

Switzerland

**Slava Rom**

USA

**Massimo Roncalli**

Italy

**Katrien Roosbroeck**

USA

**Dana Roque**

USA

**Sandrine Rorive**

Belgium

**Robert Roses**

USA

**Charles Rosser**

USA

**Giulio Rossi**

Italy

**Rossella Rota**

Italy

**Kevin Roth**

USA

**Sacha Rothschild**

Switzerland

**Etienne Rouleau**

France

**Marie-Claude Rousseau**

Canada

**Giandomenico Roviello**

Italy

**Nikoletta Rovina**

Greece

**Deodutta Roy**

USA

**Sinchita Roy-Chowdhuri**

USA

**Luca Roz**

Italy

**Kirsten Rozemeijer**

Netherlands

**Daniel Rubin**

USA

**Jordi Rubió-Casadevall**

Spain

**Eugen Ruckhäberle**

Germany

**Anja Rudolph**

Germany

**Oscar M. Rueda**

UK

**Rajesha Rupaimoole**

USA

**Rasa Ruseckaite**

Australia

**Jörn Rüssel**

Germany

**Maria Russell**

USA

**Lisa Rydén**

Sweden

**Stephen Ryder**

UK

**Mi Heon Ryu**

South Korea

**Farzaneh Sabahi**

Iran

**Sebastia Sabater**

Spain

**Renaud Sabatier**

France

**Nancy Saccone**

USA

**Laura Sadler**

UK

**Carmen Saez**

Spain

**Reza Safaralizadeh**

Iran

**Stephen Safe**

USA

**Selma Sahin**

Turkey

**Ravi Prakash Sahu**

USA

**Sei Sai**

Japan

**Pothana Saikumar**

USA

**Uksha Saini**

USA

**Pierre Saintigny**

France

**Shigehira Saji**

Japan

**Keiichi Sakai**

Japan

**Mamiko Sakata-Yanagimoto**

Japan

**Shoji Sakiyama**

Japan

**Lori Sakoda**

USA

**Yasunaru Sakuma**

Japan

**April Salama**

USA

**Mauricio Salcedo**

Mexico

**Irving Salit**

Canada

**Emmanouil Saloustros**

USA

**Frederic Saltel**

France

**Shevde Samant**

USA

**Goli Samimi**

USA

**Ayman Samkari**

USA

**Cristina Sánchez**

Spain

**Maureen Sanderson**

USA

**Milena Sant**

Italy

**Daniel Santa Mina**

Canada

**Mauro Santi**

Italy

**Sankar Sanyal**

India

**Anna Sapino**

Italy

**Gangadhara Reddy Sareddy**

USA

**Ankit Sarin**

USA

**Ana Bela Sarmento-Ribeiro**

Portugal

**Tomonori Sasahira**

Japan

**Takaaki Sasaki**

Japan

**Francesco Sassi**

Italy

**Jagannadha Sastry**

USA

**Vythialingam Sathiaseelan**

USA

**Yasufumi Sato**

Japan

**Koyanagi Satoru**

Japan

**Moira Sauane**

USA

**Sevtap Savas**

Canada

**Norie Sawada**

Japan

**Neeraj Saxena**

USA

**Okay Saydam**

Austria

**Stefania Scala**

Italy

**Aldo Scarpa**

Italy

**Matthew Schabath**

USA

**Renate Schaberl-Moser**

Austria

**Reinhold Schäfer**

Germany

**Helmut Schaider**

Australia

**Jack Schalken**

Netherlands

**Dominique Scherer**

Germany

**Hans-Ulrich Schildhaus**

Germany

**Henrik Schmidt**

Denmark

**Ralf Schmitz**

Germany

**Barbara Schneider**

USA

**Hans Schoellhammer**

USA

**Jonathan Schoenfeld**

USA

**Nathalie Scholler**

USA

**Mario Schootman**

USA

**Andres Jan Schrader**

Germany

**Peter Schraml**

Switzerland

**Markus Schuler**

Germany

**Hildegard M. Schuller**

USA

**Beate Schultheis**

Germany

**Wolfgang Schulz**

Germany

**Claudia Schuurhuizen**

Netherlands

**Matthias Schwab**

Germany

**Edward Schwalbe**

UK

**Theresa Schwartz**

USA

**Judith Schwartzbaum**

USA

**Stephen Schwarz**

USA

**Thomas Schwarzbraun**

Austria

**Lukas Schwentner**

Germany

**Jennifer Scott**

Australia

**Anna Ivana Scovassi**

Italy

**Martin Scurr**

UK

**Susanne Sebens**

Germany

**Michael Seckl**

UK

**Beatrice Seddon**

UK

**Violaine See**

UK

**Veronika Seebacher**

Austria

**Amikar Sehdev**

USA

**Silvia Selinski**

Germany

**Luke Selth**

Australia

**Lorenzo Sempere**

USA

**Thomas Semrad**

USA

**Sharmila Sengupta**

India

**Carlo Senore**

Italy

**Marialuisa Sensi**

Italy

**Theodoros N. Sergentanis**

Greece

**Zineb Serhier**

Morocco

**Massimo Serra**

Italy

**Diego Serraino**

Italy

**Fausto Sessa**

Italy

**Vijay Setaluri**

USA

**Bhuvana Setty**

USA

**Alberto Severini**

Canada

**Patricia Severino**

Brazil

**Victoria Shaffer**

USA

**Walayat Shah**

Pakistan

**Safi Shahda**

USA

**Jorida Shahinas**

Albania

**Mohammad Shakhatreh**

USA

**Ge Shan**

China

**Mala Shanmugam**

USA

**Jian-Yong Shao**

China

**Jeremy Shapiro**

Australia

**Sue Shapses**

USA

**Shashwat Sharad**

USA

**Daya Nand Sharma**

India

**Jacqueline Shaw**

UK

**Carrie Shawber**

USA

**Jin-Xiong She**

USA

**Fatma Shebl**

USA

**Umer Sheikh**

USA

**Orla Sheils**

Ireland

**Rachel Shelton**

USA

**Yaxing Shen**

China

**Trevor Shepherd**

Canada

**Yufang Shi**

China

**Hiroaki Shiba**

Japan

**Toshio Shimizu**

Japan

**Aesun Shin South**

Korea

**Masaki Shiota**

Japan

**Lawrence Shirley**

USA

**Mark Shiroishi**

USA

**Neelam Shirsat**

India

**Yow-Ling Shiue**

Taiwan

**Maya Shmulevitz**

Canada

**Lillie Shockney**

USA

**Susan Short**

UK

**Maria Shoshan**

Sweden

**Louise Showe**

USA

**Stuti Shroff**

USA

**Surendra Shukla**

USA

**Andrew Shuman**

USA

**Miroslawa Siatecka**

Poland

**David Sibson**

UK

**Jason Sicklick**

USA

**Anna Sidorchuk**

Sweden

**Christian Siebel**

USA

**Peter Siegel**

Canada

**Markus Siegelin**

USA

**Britta Siegmund**

Germany

**Cornelis Sier**

Netherlands

**Anieta Sieuwerts**

Netherlands

**William Sikov**

USA

**Dan-Arin Silasi**

USA

**Andrew Silver**

UK

**Michael Simon**

USA

**Charles Simone**

USA

**George Simos**

Greece

**Andrew Sims**

UK

**Tomas Simunek**

Czech Republic

**Marko Simunovic**

Canada

**Josef Singer**

Austria

**Sheila Singh**

Canada

**Aatur Singhi**

USA

**Prem Sinha**

USA

**Debora Sinner**

USA

**Terence Sio**

USA

**Ronit Sionov**

Israel

**Diego Sisci**

Italy

**Tristan Sissung**

USA

**Freddy Sitas**

Australia

**Dongrong Situ**

China

**Jan Konrad Siwicki**

Poland

**Stefan Siwko**

USA

**Arvid Sjölander**

Sweden

**Melissa Skala**

USA

**Alena Skalova**

Czech Republic

**Tina Skinner**

Australia

**Joseph Skitzki**

USA

**Mariusz Skwarczynski**

Australia

**Loki Skylizard**

USA

**Tania Slatter**

New Zealand

**J. Mark Sloan**

USA

**Alexandra Smith**

UK

**Winnie So**

China

**Tomotaka Sobue**

Japan

**Anastasios Sofiadis**

Sweden

**Eric Solary**

France

**Flavio Solca**

Austria

**Maria Soldatkina**

Ukraine

**Hosanna Soler-Vila**

Spain

**Alex Soltermann**

Switzerland

**Gunhild Sommer**

USA

**Jianguo Song**

China

**Geoffrey Sonn**

USA

**Kenzo Sonoda**

Japan

**Guru Sonpavde**

USA

**Pierre Sonveaux**

Belgium

**Yu Yang Soon**

Singapore

**Halfdan Sorbye**

Norway

**Robert Soslow**

USA

**José Luis Soto**

Spain

**Joseph Sottnik**

USA

**Andrea Sottoriva**

UK

**Michael Soulen**

USA

**Nor Eddine Sounni**

Belgium

**Silvia Sousa**

Brazil

**Christopher South**

USA

**Mohammed Soutto**

USA

**Martin Spahn**

Switzerland

**Melanie Spears**

Canada

**Hanno Specht**

Germany

**Nathalie Sphyris**

UK

**Claudio Spick**

Austria

**Michael Spiotto**

USA

**Carlo Spirli**

USA

**Gaya Spolverato**

USA

**Christoph Springfeld**

Germany

**Jeremy Squire**

Brazil

**Aswathy Sreedevi**

India

**Gokul Sridharan**

India

**Padmanabhan Srinivasan**

USA

**Sanjeeva Srivastava**

India

**Eri Srivatsan**

USA

**Kalkunte Srivenugopal**

USA

**Caryn St. Clair**

USA

**Elvira Stacher**

Austria

**Teresa Stachowicz-Stencel**

Poland

**Venturina Stagni**

Italy

**Olle Stål**

Sweden

**Frank Stanczyk**

USA

**Rachel Stankowski**

USA

**Sasha Stanton**

USA

**Keith Stantz**

USA

**Jerod Stapleton**

USA

**Mitchell Stark**

Australia

**Patrick Starlinger**

Austria

**Maria Stasiuk**

Poland

**Giorgio Stassi**

Italy

**Lucy Stead**

UK

**Jason Steel**

USA

**Andrew Steele**

UK

**Barbara Stefanska**

USA

**Felix Steger**

Germany

**Alexander Stegh**

USA

**Alexander Stein**

Germany

**Eric Steiner**

Germany

**Diana Steinmann**

Germany

**Jan Stenvang**

Denmark

**Julie Sterling**

USA

**Peter Stern**

UK

**Christopher Stevenson**

Australia

**David Stewart**

Canada

**Sebastian Stintzing**

Germany

**Lucia Anna Stivala**

Italy

**Christian Stock**

Germany

**Dick Stockelberg**

Sweden

**Friedrich Stoelzel**

Germany

**Gustavo Stolovitzky**

USA

**Walter Storkus**

USA

**Fabio Stossi**

USA

**Christos Stournaras**

Greece

**Jonathan Strefford**

UK

**Thomas Stricker**

USA

**John Strickler**

USA

**Staffan Strömblad**

Sweden

**Tanja Stueber**

Germany

**Kai Stühler**

Germany

**Dwayne Stupack**

USA

**Emelie Styring**

Sweden

**Shicheng Su**

China

**Visvaraja Subrayan**

Malaysia

**Sunil Sudarshan**

USA

**Kazuki Sugahara**

USA

**Guangchao Sui**

USA

**Rudy Suidan**

USA

**Saraswati Sukumar**

USA

**Ryan Sullivan**

USA

**Vadim Sumbayev**

UK

**Matt Summers**

USA

**Ying-Shi Sun**

China

**Eric Sundberg**

USA

**Karin Sundfeldt**

Sweden

**Rajah Supramaniam**

Australia

**Sripathi Sureban**

USA

**Colleen Sweeney**

USA

**Peter Sykes**

New Zealand

**Argiris Symeonidis**

Greece

**Ildiko Szabo**

Italy

**Zoltan Szallasi**

USA

**Joanna Szkandera**

Austria

**Russell Szmulewitz**

USA

**Takahiro Tabuchi**

Japan

**Keiichiro Tada**

Japan

**Kyung Tae**

South Korea

**Scott Tagawa**

USA

**Alberto Tagliafico**

Italy

**Jean-Bosco Tagne**

USA

**Ayumu Taguchi**

USA

**Alain Taieb**

France

**Tatsuro Tajiri**

Japan

**Mamoru Takada**

USA

**Naohisa Takaoka**

Japan

**Atsuo Takashima**

Japan

**Kazuki Takeishi**

Japan

**Fumitaka Takeshita**

Japan

**Nagio Takigawa**

Japan

**Giampaolo Talamo**

USA

**Evelyn Talbott**

USA

**Wayne Tam**

USA

**Elena Tamborini**

Italy

**Rulla May Tamimi**

USA

**Teruko Tamura**

Germany

**Benjamin Tan**

USA

**Motoyoshi Tanaka**

Japan

**Carol Tang**

Singapore

**Michael Tangrea**

USA

**Kandice Tanner**

USA

**Tamara Tanos**

Italy

**Lu Tao**

USA

**Coya Tapia**

USA

**Corrado Tarella**

Italy

**Line Tarpgaard**

Denmark

**Teresa Tarrant**

USA

**Claudio Tatsui**

USA

**Yoshihiro Tatsumi**

Japan

**Eliane Tabea Taube**

Germany

**Michele Tavecchio**

USA

**Daniela Taverna**

Italy

**Molly Taylor**

UK

**Julia Tchou**

USA

**Muy-Kheng M. Tea**

Austria

**Andrew Tee**

UK

**Tomas Tefera**

Ethiopia

**Clemens Tempfer**

Germany

**Sally Temraz**

Lebanon

**Kevin Ten Haaf**

Netherlands

**Jason Tennessen**

USA

**Daniela Terracciano**

Italy

**Paul Terry**

USA

**Anna Tessari**

USA

**Traci Testerman**

USA

**Michael Tetzlaff**

USA

**Bjoern Tews**

Germany

**Andreas Thalheimer**

Germany

**Raynoo Thanan**

Japan

**Gita Thanarajasingam**

USA

**Ekkasit Tharavichitkul**

Thailand

**Montarat Thavorncharoensap**

Thailand

**Sue-Mian Then**

Malaysia

**Kullathorn Thephamongkhol**

Thailand

**Frank Thevenod**

Germany

**Sam Thiagalingam**

USA

**Minta Thomas**

Belgium

**Cheryl Thompson**

USA

**Hauke Thomsen**

Germany

**Ebbe Thorgersen**

Norway

**Vesteinn Thorsson**

USA

**Peter Thuss-Patience**

Germany

**Wenjing Tian**

China

**Maarit Tiirikainen**

USA

**Nikolai Timchenko**

USA

**Paul Timpson**

Australia

**Dina Tiniakos**

UK

**Fabricio Tinôco Alvim De Souza**

Brazil

**Carlos Tirado**

USA

**Venkataswarup Tiriveedhi**

USA

**Nina Tirnitz-Parker**

Australia

**Jasmin Tiro**

USA

**Pankaj Tiwari**

USA

**Katherine Tkaczuk**

USA

**Tilman Todenhöfer**

Germany

**Sebastien Toffoli**

Belgium

**Eriko Tohno**

Japan

**Masakazu Toi**

Japan

**Anna-Maria Tokes**

Hungary

**Trygve Tollefsbol**

USA

**Dhanendra Tomar**

USA

**Atsushi Tomioka**

Japan

**Masaki Tomita**

Japan

**Yokota Tomoya**

Japan

**Steven Toms**

USA

**Ciprian Tomuleasa**

Romania

**Alicia Tone**

Canada

**Yeqing Tong**

China

**Catherine Too**

Canada

**Baki Topal**

Belgium

**Bastiaan Tops**

Netherlands

**Ruurd Torensma**

Netherlands

**Toshihiko Torigoe**

Japan

**Adetunji Toriola**

USA

**Luigi Tornillo**

Switzerland

**Sofia Torres**

Canada

**Moriwaki Toshikazu**

Japan

**Cyril Touboul**

France

**Manatomo Toyono**

Japan

**Britton Trabert**

USA

**Nirupma Trehanpati**

India

**Elisa Trevisan**

Italy

**Gaurav Tripathi**

Canada

**Tiziana Triulzi**

Italy

**Meghana Trivedi**

USA

**Grant Trobridge**

USA

**Jaroslav Truksa**

Czech Republic

**Robert Trumbly**

USA

**Matthew Truong**

USA

**Volodymyr Tryndyak**

USA

**Sen-Tien Tsai**

Taiwan

**Chris Tselepis**

UK

**Yau-Lin Tseng**

Taiwan

**Konstantinos Tsilidis**

Greece

**George Tsirakis**

Greece

**Junji Tsurutani**

Japan

**Yaping Tu**

USA

**Tugba Tumer**

Turkey

**Krzysztof Tupikowski**

Poland

**Kemal Turhan**

Turkey

**Alice Turner**

UK

**Jonathan Tward**

USA

**Chris Twelves**

USA

**Ethika Tyagi**

USA

**Paolo Ubezio**

Italy

**Enoch Uche**

Nigeria

**Emeka Udeh**

Nigeria

**Keisuke Uehara**

Japan

**Takashi Ueki**

Japan

**Naoto Ueno**

USA

**Tsukuru Umemura**

Japan

**Esther Una Cidon**

UK

**Ghanshyam Upadhyay**

India

**Ruchan Uslu**

Turkey

**Ca Uyl-De Groot**

Netherlands

**Ratna Vadlamudi**

USA

**Milind Vaidya**

India

**Vladimir Valakh**

USA

**Fatima Valdes Mora**

Australia

**Nuno Vale**

Portugal

**Daniel Vallbohmer**

Germany

**Adamo Valle**

Spain

**Elske Van Den Akker**

Netherlands

**Adriaan Van Den Brule**

Netherlands

**Donald L. Van Der Peet**

Netherlands

**Marjolein Van Der Poel**

Netherlands

**Nadia Van Der Spek**

Netherlands

**Paul Van Diest**

Netherlands

**Thorbald Van Hall**

Netherlands

**Guy Van Hazel**

Australia

**Mieke Van Hemelrijck**

UK

**Folkert Van Kemenade**

Netherlands

**Brian Van Ness**

USA

**Nicolien Van Ravesteyn**

Netherlands

**Joost Van Rosmalen**

Netherlands

**Brian Van Tine**

USA

**Kimberly Van Zee**

USA

**Christopher Vandenbussche**

USA

**Kate Vandyke**

Australia

**Sakari Vanharanta**

UK

**John Varlotto**

USA

**Vasyl Vasko**

USA

**Erik Vassella**

Switzerland

**Naveen Vasudev**

UK

**Cecily Vaughn**

USA

**Guillermo Velasco**

Spain

**Guermarie Velazquez Torres**

USA

**Kiran Velpula**

USA

**Roberta Venturella**

Italy

**Gyorgy Veress**

Hungary

**Daniele Vergara**

Italy

**Benjamin Verillaud**

France

**Raj Kumar Verma**

USA

**Henri Versteeg**

Netherlands

**Claude Vezeau**

Canada

**Massimo Vicentini**

Italy

**Patrizia Vici**

Italy

**Andrew Vickers**

USA

**Daniel Vidal**

Brazil

**Andre F Vieira**

Portugal

**Eduardo Vilar Sanchez**

USA

**Reina Villareal**

USA

**Bruno Vincenzi**

Italy

**Maria Vinci**

UK

**Nerissa Viola-Villegas**

USA

**Pablo Vivas-Mejia**

USA

**Erina Vlashi**

USA

**Ulla Vogel**

Denmark

**Ioannis Voutsadakis**

Canada

**Semir Vranic Bosnia And**

Herzegovina

**Te Vuong**

Canada

**Mathilde Wagner**

France

**Hiroki Wakabayashi**

Japan

**Bruce Walcheck**

USA

**Jens Waldmann**

Germany

**Logan Walker**

New Zealand

**Kristin Wallace**

USA

**John Wallbillich**

USA

**Tonya Walser**

USA

**Siun Walsh**

Ireland

**Axel Walther**

UK

**Guohui Wan**

USA

**Ya-Wen Wang**

China

**Andrea Wang-Gillam**

USA

**Nissar Ahmad Wani**

USA

**Erica Warner**

USA

**Graham Warren**

USA

**Agata Wasik**

Sweden

**Naoki Watanabe**

Japan

**Joshua Waters**

USA

**Ian Watson**

Canada

**Tonya Webb**

USA

**Dan Wechsler**

USA

**Daniel Weekes**

UK

**Taotao Wei**

China

**Wei Gong Wei Gong**

China

**Stefan Weiler**

Switzerland

**Jeffrey Weinberg**

USA

**Sheila Weinmann**

USA

**Geoffrey R. Weiss**

USA

**Meng Welliver**

USA

**Jing-Ru Weng**

Taiwan

**Haim Werner**

Israel

**Robert Wesolowski**

USA

**Robert West**

UK

**Kelly Whelan**

USA

**Patsy Whelehan**

UK

**Noel Whitaker**

Australia

**Jeffrey White**

USA

**Becky Whiteman**

UK

**Max Wicha**

USA

**Edyta Wieczorek**

Poland

**Armin Wiegering**

Germany

**Malgorzata Wiench**

UK

**Alan Wigg**

Australia

**Tanya Wildes**

USA

**Anne Wilkinson**

Australia

**Peer Wille-Jorgensen**

Denmark

**Stefan Willems**

Netherlands

**Basem William**

USA

**Christopher Williams**

USA

**Sean Williamson**

USA

**David Wilson**

USA

**Saskia Wilting**

Netherlands

**Jay Wimalasena**

Sri Lanka

**Robert Winn**

USA

**Trisha Wise-Draper**

USA

**Bea Wisman**

Netherlands

**Michel Wissing**

Netherlands

**Josephine Wixted**

USA

**Maciej Wnuk**

Poland

**Philipp Wolf**

Germany

**Anita Wolfer**

Switzerland

**Hendrik Andreas Wolff**

Germany

**Aaron H. Wolfson**

USA

**Young-Joo Won**

South Korea

**Stephen Wong**

Australia

**Yanghee Woo**

USA

**Terri Wood**

USA

**Terri L. Woodard**

USA

**Michael Worley**

USA

**Thomas Worst**

Germany

**Antoinette Wozniak**

USA

**Curtis Wray**

USA

**Olivia Wright**

Australia

**Wei Wu**

Canada

**Bs Wung**

Taiwan

**Lynda Wyld**

UK

**Jose Candido Xavier Junior**

Brazil

**Jialing Xiang**

USA

**Qiang Xiao**

China

**Yang Xie**

USA

**Mingzhao Xing**

USA

**Jiejie Xu**

China

**Umesh Yadav**

India

**Hideki Yagi**

Japan

**Vasily Yakovlev**

USA

**Bulent Yalcin**

Turkey

**Sohsuke Yamada**

Japan

**Hiromasa Yamamoto**

Japan

**Hiroharu Yamashita**

Japan

**Shinichi Yamauchi**

Japan

**Hiroki Yamaue**

Japan

**Naoki Yanagawa**

Japan

**Ryu Yanagisawa**

Japan

**Nozomu Yanaihara**

Japan

**Burton Yang**

Canada

**Jeffrey Yap**

USA

**Lei Ye**

China

**Chia Jui Yen**

Taiwan

**Jim Yeung**

USA

**Ho-Young Yhim**

South Korea

**Yajun Yi**

USA

**John Yim**

USA

**Jun Yin**

China

**Hou Yingyong**

China

**Michele Yip-Schneider**

USA

**Tomoya Yokota**

Japan

**Raymund Yong**

USA

**Bongin Yoo**

USA

**Kyong-Ah Yoon**

South Korea

**Kenji Yorita**

Japan

**Akira Yoshida**

Japan

**Kosuke Yoshihara**

Japan

**Noritada Yoshikawa**

Japan

**Nobuyasu Yoshimoto**

Japan

**Shigefumi Yoshino**

Japan

**Hironori Yoshiyama**

Japan

**Tiangeng You**

China

**Danny Youlden**

Australia

**Robert P. Young**

New Zealand

**Jack Youngren**

USA

**Luoting Yu**

China

**Xianglin Yuan**

China

**Lu Yue**

China

**Fiona Yull**

USA

**Nabiha Yusuf**

USA

**Brian Zabel**

USA

**Eldad Zacksenhaus**

Canada

**Peter Zage**

USA

**Per-Henrik Zahl**

Norway

**Raza Zaidi**

USA

**Ian Zajac**

Australia

**Claudio Zamagni**

Italy

**Xingxing Zang**

USA

**Uwe Zangemeister-Wittke**

Switzerland

**Rafael Zanin**

Brazil

**Antonella Zannetti**

Italy

**Apostolos Zaravinos**

Sweden

**Hajo Zeeb**

Germany

**Amer Zeidan**

USA

**Charnita Zeigler-Johnson**

USA

**Robert Zeillinger**

Austria

**Alain Zeimet**

Austria

**Anthony G. Zeitouni**

Canada

**Steven Zeliadt**

USA

**Hitoshi Zembutsu**

Japan

**Maryam Zenali**

USA

**Tao Zeng**

China

**Han Zhang**

Canada

**Chenghai Zhao**

China

**Hua-Chuan Zheng**

China

**Zhao Zhiming**

China

**Yuan Zhiyong**

China

**Li Zhong**

USA

**Xiaoying Zhou**

China

**Jiaan Zhu**

China

**Mel Ziman**

Australia

**Tim Zimmermann**

Germany

**Ralph Zinner**

USA

**Oliver Zivanovic**

USA

**Inti Zlobec**

Switzerland

**Gabriele Zoppoli**

Italy

**Ellen Zwarthoff**

Netherlands

**Matjaz Zwitter**

Slovenia

